# Bacteria related to tick-borne pathogen assemblages in *Ornithodoros* cf. *hasei* (Acari: Argasidae) and blood of the wild mammal hosts in the Orinoquia region, Colombia

**DOI:** 10.1007/s10493-022-00724-9

**Published:** 2022-07-13

**Authors:** Juan D. Carvajal-Agudelo, Héctor E. Ramírez-Chaves, Paula A. Ossa-López, Fredy A. Rivera-Páez

**Affiliations:** 1 Grupo de Investigación en Genética, Biodiversidad y Manejo de Ecosistemas (GEBIOME), Departamento de Ciencias Biológicas, Facultad de Ciencias Exactas y Naturales, Universidad de Caldas, Calle 65 N° 26-10, 170004 Manizales, Caldas Colombia; 2 Centro de Museos, Museo de Historia Natural, Universidad de Caldas, Calle 65 N° 26-10, 170004 Manizales, Caldas Colombia; 3 Doctorado en Ciencias, Biología, Facultad de Ciencias Exactas y Naturales, Universidad de Caldas, Calle 65 No. 26-10, 170004 Manizales, Caldas Colombia

**Keywords:** *Borrelia*, Chiroptera, Endosymbiont, Microbiome, Soft tick, *Rickettsia*

## Abstract

Interest in research on soft ticks has increased in recent decades, leading to valuable insight into their role as disease vectors. The use of metagenomics-based analyses have helped to elucidate ecological factors involved in pathogen, vector, and host dynamics. To understand the main bacterial assemblages present in *Ornithodoros* cf. *hasei* and its mammalian hosts, 84 ticks and 13 blood samples from bat hosts (Chiroptera) were selected, and the 16S rRNA gene V4 region was sequenced in five pools (each one related to each host-tick pairing). Bacterial taxonomic assignment analyses were performed by comparing operational taxonomic units (OTUs) shared between ticks and their host blood. This analysis showed the presence of Proteobacteria (38.8%), Enterobacteriaceae (25%), Firmicutes (12.3%), and Actinobacteria (10.9%) within blood samples, and Rickettsiaceae (39%), Firmicutes (25%), Actinobacteria (13.1%), and Proteobacteria (9%) within ticks. Species related to potentially pathogenic genera were detected in ticks, such as *Borrelia* sp., *Bartonella tamiae*, *Ehrlichia* sp. and *Rickettsia*-like endosymbiont, and the presence of these organisms was found in all analyzed bat species (*Cynomops planirostris*, *Molossus pretiosus*, *Noctilio albiventris*), and *O.* cf. *hasei*. About 41–48.6% of bacterial OTUs (genera and species) were shared between ticks and the blood of bat hosts. Targeted metagenomic screening techniques allowed the detection of tick-associated pathogens for *O.* cf. *hasei* and small mammals for the first time, enabling future research on many of these pathogens.

**Supplementary Information:**

The online version contains supplementary material available at 10.1007/s10493-022-00724-9.

## Introduction

Globally, ticks comprise about 955 species from three families: Ixodidae (ca. 736 species), Argasidae (ca. 218 species) and Nuttalliellidae (1 species), and about a quarter of all species are found within the Neotropical region (Dantas-Torres et al. 2019). Common methods to investigate the role of ticks as a disease vector involve DNA-based diagnostic molecular methods (i.e., PCR-based), but these methods alone are unable to provide insight into other ecological factors (e.g., transmission of organisms between tick species and hosts, detection of other microorganisms, parasite loads, etc. (Estrada-Peña et al. 2013; Tijsse-Klasen et al. 2014; Cabezas-Cruz et al. 2018). Targeted microbiome analysis enables the detection and identification of bacteria assemblages by metagenomic profiling (Cabezas-Cruz et al. 2018; Greay et al. 2018; Thoendel 2020) and has allowed for increased detection of new microorganism species, strains, and genetic variation within ticks (Tokarz and Lipkin 2021). The list of potential tick-borne pathogens is growing (Shapiro et al. 2010; Subramanian et al. 2012; Wu-Chuang et al. 2021) and microbiome studies are beginning to take on a critical role in the functional investigation of microbial communities; this is largely due to the understanding of vector-borne diseases in relation to their pathogenicity, ecology, reproduction, potential hosts, and the implication in human and animal public health (Vilcins et al. 2009; Rynkiewicz et al. 2015; Bonnet et al. 2017). For hard ticks from the genus *Amblyomma* (*A. americanum*, *A. maculatum*, *A. tuberculatum*), *Ixodes ricinus*, and *Rhipicephalus microplus*, microbiome analyses have been successfully used to characterize bacterial communities involving pathogenic bacteria species such as *Borrelia*, *Anaplasma*, *Rickettsia* and *Ehrlichia* (Andreotti et al. 2011; Carpi et al. 2011; Menchaca et al. 2013; Budachetri et al. 2014, 2016; Wu-Chuang et al. 2021), whereas other studies have focused on complete pathobiome analysis involving ticks (Vayssier-Taussat et al. 2015; Bennett 2017; Zhuang et al. 2018; Tufts et al. 2020). Similarly, research on soft ticks (Argasidae) has gained importance in recent decades and has provided considerable information on the ecology, taxonomy, systematics, and their role as vectors of pathogens (Wen and Chen 2016; Nava et al. 2017). Nonetheless, studies focusing on bacterial assemblages of soft ticks are known for limited species such as *Argas japonicus* with special interest in pathogens such as *Rickettsia* (Yan et al. 2019); *Ornithodoros muesebecki* with interest in pathogenic groups such as *Borrelia*, *Coxiella*, and *Rickettsia* (Alkayyoomi 2018); and *Ornithodoros turicata* and its general microbiome (Barraza-Guerrero et al. 2020). In Latin America, studies on soft ticks have focused on the role as vectors of *Anaplasma*, *Borrelia*, and *Rickettsia* (Loftis et al. 2005; Tahir et al. 2016; Muñoz-Leal et al. 2019; Luz et al. 2019; de Oliveira et al. 2020). Nonetheless, other epidemiological groups of argasid-related organisms such as *Coxiella*, responsible for Crimean-Congo hemorrhagic fever, West Nile virus, and Royal Farm virus, have re-emerged as common pathogens (Manzano-Román et al. 2012; Sarwar 2017; Diaz 2021; Hanafi-Bojd et al. 2021; Kazim et al. 2021).

In Colombia, studies involving ticks, bacterial assemblages and pathogens related to soft ticks are incipient and fragmented. Within the 58 tick species found, 51 are associated with wild mammals Hidalgo et al. 2011; Esser et al. 2016; Faccini-Martínez et al. 2016; Rivera-Páez et al. 2018a; Guglielmone 2021; Ortíz-Giraldo et al. 2021): 43 of these species belong to Ixodidae and 15 to Argasidae. For the latter, 12 species (*Antricola mexicanus* and 11 species of the genus *Ornithodoros*: *O. azteci*, *O. brodyi*, *O hasei*, *O. marinkellei*, *O. marmosae*, *O. peropteryx*, *O. puertoricensis*, *O. rossi*, *O. rudis*, *O. talaje* and *O. yumatensis*) are related to mammals (Ortíz-Giraldo et al. 2021). Some species distributed in Colombia such as *O. rudis* have been related to recurrent fevers in the 20th century (Franco et al. 1911; Pino Pou 1984; Faccini-Martínez and Botero-García 2016) but the old and rare reports of *Ornithodoros* spp. require taxonomic confirmation (López et al. 2021).

In particular, *Ornithodoros hasei* (Schulze), is one of the most widely distributed soft ticks in South America and is present in about 20 countries (Nava et al. 2017) where it has been primarily associated with bats (Marinkelle and Grose 1981; Ortíz-Giraldo et al. 2021). Molecular detection of *Rickettsia* has been verified in *O. hasei* in Argentina (Colombo et al. 2020), French Guyana (Tahir et al. 2016) and *Borrelia* in Brazil (Muñoz-Leal et al. 2021a). In Colombia, *O. hasei* has been recorded mainly in bats (Marinkelle and Grose 1981; Tarquino-Carbonell et al. 2015; Ortíz-Giraldo et al. 2021); however, to date there are no reports of the presence of associated pathogens. Given this, the objective of this study is to describe the main bacterial and the related to tick-borne pathogens assemblages present in *Ornithodoros* cf. *hasei* and its mammalian hosts in the Orinoquia region of Colombia.

## Materials and methods

### Collection of samples and identification of specimens

Samples were obtained in the municipalities of Arauca, Cravo Norte, Tame, Department of Arauca (Orinoquia region of Colombia), between November and December 2018, and March, July, and August 2019 (Table S1). Bats (Chiroptera) were captured and sampled using standard protocols (Bazán-León 2011). The collected ticks were stored in 2-mL Eppendorf tubes with 96% ethanol. Blood samples were taken from mammal samples hosting argasids via axillary venipuncture, and deposited in 5-mL heparinized tubes mixed at a 1:9 ratios with DNA/RNA Shield reagent (Zymo Research, Irvine, CA, USA) according to the manufacturer’s instructions and stored at −80 °C.

Sample collection was conducted under the framework permit granted by the National Environmental Licensing Authority (ANLA) to the Universidad de Caldas as stipulated in resolution 02497 of December 31, 2018. Additionally, no species registered in the list of threatened wild species of Colombian biological diversity consigned in resolution nr. 1912 of 2017 were collected. All samples and specimens collected were deposited in the mammal collection of the Museum of Natural History of the Universidad de Caldas (MHN-UCa), and identified using taxonomic keys (e.g., Gardner 2008).

Morphological identification of soft ticks to species level was performed following clarification in 25% KOH and fixation in Hoyer medium (Muñoz-Leal et al. 2019). The dichotomous keys of Filippova (1966), Hoogstraal (1985), Klompen and Oliver (1993), Camicas et al. (1998) and Battesti et al. (2006) were used. After morphological identification, individuals were processed for DNA extraction using the DNeasy Blood and Tissue kit (Qiagen) according to the manufacturer’s suggested protocol. DNA extracted from each tick was amplified via PCR targeting an approximate 460 bp fragment corresponding to the mitochondrial 16S rDNA gene using primers F 5′-CCG GTC TGA ACT CAG ATC AAG-3′ and R 5′-GCT CAA TGA TTT TTT AAA TTG CTG-3′ (Mangold et al. 1998). The amplicons were used for Sanger sequencing at Macrogen (Seoul, South Korea). Confirmation of soft tick species was performed using BLAST and comparison of a maximum likelihood (ML) similarity analysis with 1000 iterations in MEGA X and a Bayesian (BY) analysis using MrBayes v.3.2.7 (Ronquist et al. 2012) via CIPRES tool (Miller et al. 2011), employing four independent Markov chains, 15,000000 generations and sampling every 1000 generations. The first 25% of the trees were discarded and the remaining trees were used to calculate posterior probability values. The trees were edited using the iTOL tool (Letunic and Bork 2019). Identification and analysis of ticks were performed based on similarity comparisons with public sequences in GenBank and BOLD (Barcode of Life Data Systems) databases. The sequences obtained in this study were deposited in GenBank (accessions MZ773894–MZ773899).

### Sample selection, preparation, and sequencing

All ticks selected were attached to the hosts (feeding stage). For tick pools, a selection was made on the abundance of ticks found on the hosts, and only the samples that contained ticks and the mammalian blood collected on RNA/DNA-shield were used. This selection included five pools of ticks, as well as blood pools from bat species such as *Cynomops planirostris*, *Molossus pretiosus*, *Myotis handleyi*, and two pools from *Noctilio albiventris* (Table 1). Ticks were sterilized using 1% sodium hypochlorite followed by washes with 70% ethanol and distilled water (Binetruy et al. 2019). DNA from ticks and blood of wild mammals was obtained using ZymoBIOMICS DNA/RNA Miniprep Kit (Zymo Research), according to the specific instructions that involve the maceration of all samples through bead beating (30 min), and specifically for blood, 750 µL of sampled blood (blood + RNA/DNA shield) were used to the whole process according to the specific instructions. Samples were prepared in argasid and mammalian blood pools. Samples were sent for sequencing to amplify the V4 region of the 16S gene (ca. 250 bp) bacterial rRNA (fusion primers/515F-806R) using the targeted sequencing service at BGI Genomics (Hong Kong, China). This includes quality control to verify the viability of the sequencing process, 16S gene library preparation and use of the Illumina HiSeq2500 sequencing platform to obtain amplified reads.

**Table 1:** Conformation of the pools used in the study for *Ornithodoros* cf*. hasei* ticks and blood from wild mammals of Arauca, Orinoquia region, Colombia (all ticks used in the study were in the larval stage)

Pool ID	Museum code	No. ticks	Host	Locality	Coordinates
3AS	MHN-UCa-M 2808	1	*Molossus pretiosus*	Arauca, Vereda El Socorro, Finca Los Trompillos	06°47′3.23" N 70°42′8.2" W
	MHN-UCa-M 2863	1	*Molossus pretiosus*		
	MHN-UCa-M 2822	10	*Molossus pretiosus*		
	MHN-UCa-M 2869	2	*Molossus pretiosus*		
5AS	MHN-UCa-M 2327	1	*Cynomops planirostris*	Cravo Norte, Vereda El Deleite	06°32′25.2" N 70°31′23.6" W
	MHN-UCa-M 2328	1	*Cynomops planirostris*		
	MHN-UCa-M 2317	1	*Cynomops planirostris*	Arauca, Vereda Las Plumas, Sitio Los Cunaguaros	06°36′15" N 70°29′52" W
	MHN-UCa-M 2323	1	*Cynomops planirostris*		
6AS*	MHN-UCa-M 2265	3	*Noctilio albiventris*	Cravo Norte, Vereda El Deleite	06°32′25.2" N 70°31′23.6" W
	MHN-UCa-M 2253	6	*Noctilio albiventris*		
	MHN-UCa-M 2262	1	*Noctilio albiventris*	Arauca, Vereda Las Plumas, Sector Guayabital	06°37′29.4" N 70°35′9.9" W
7AS	MHN-UCa-M 2806	50	*Noctilio albiventris*	Arauca, Vereda El Socorro, Finca Los Trompillos	06°47′3.23" N 70°42′8.2" W
8AS	MHN-UCa-M 2928	6	*Myotis handleyi*	Arauca, Campus Universidad Nacional Sede Orinoquía, Vereda Mategallina, road to Caño Limón	07°0′8.47" N 70°44′44.2" W

*The 6SAN pool belongs to the *N. albiventris* marked in all the document as *

### Bioinformatics analysis

Sequences were individually filtered to obtain high quality clean sequences using fqtools software; fqcheck (v.0.25), readfq (v.1.0) (removal of truncated reads and reads below 75% length) (Fadrosh et al. 2014; Droop 2016); and cutadapt (v.2.6) (Martin 2011), to remove reads contaminated with adapter sequences, ambiguous bases (N bases), and low complexity. Sequence consensus of paired-end reads was performed using FLASH v.1.2.11 (Magoč and Salzberg 2011). Clustering, chimera removal, rarefaction curves, Shannon index, and sequence identification at 97% identity was performed in QIIME 2 (v.2021.04) (Estaki et al. 2020). Operational taxonomic units (OTUs) were generated using the VSEARCH tool (Rognes et al. 2016) and the Greengenes database (Kaehler et al. 2019). The MEGAN v4 program was used to compare the readings with NCBI (Huson and Mitra, 2012). To relate the shared bacterial communities between ticks and hosts, a Venn diagram was made using the taxonomic classification of OTUs (genus and species) by means of the vegan package (Oksanen et al. 2013), in the R v.4.0 program. Sequences are available under the BioProject ID PRJNA767818.

## Results

**Fig. 1:**
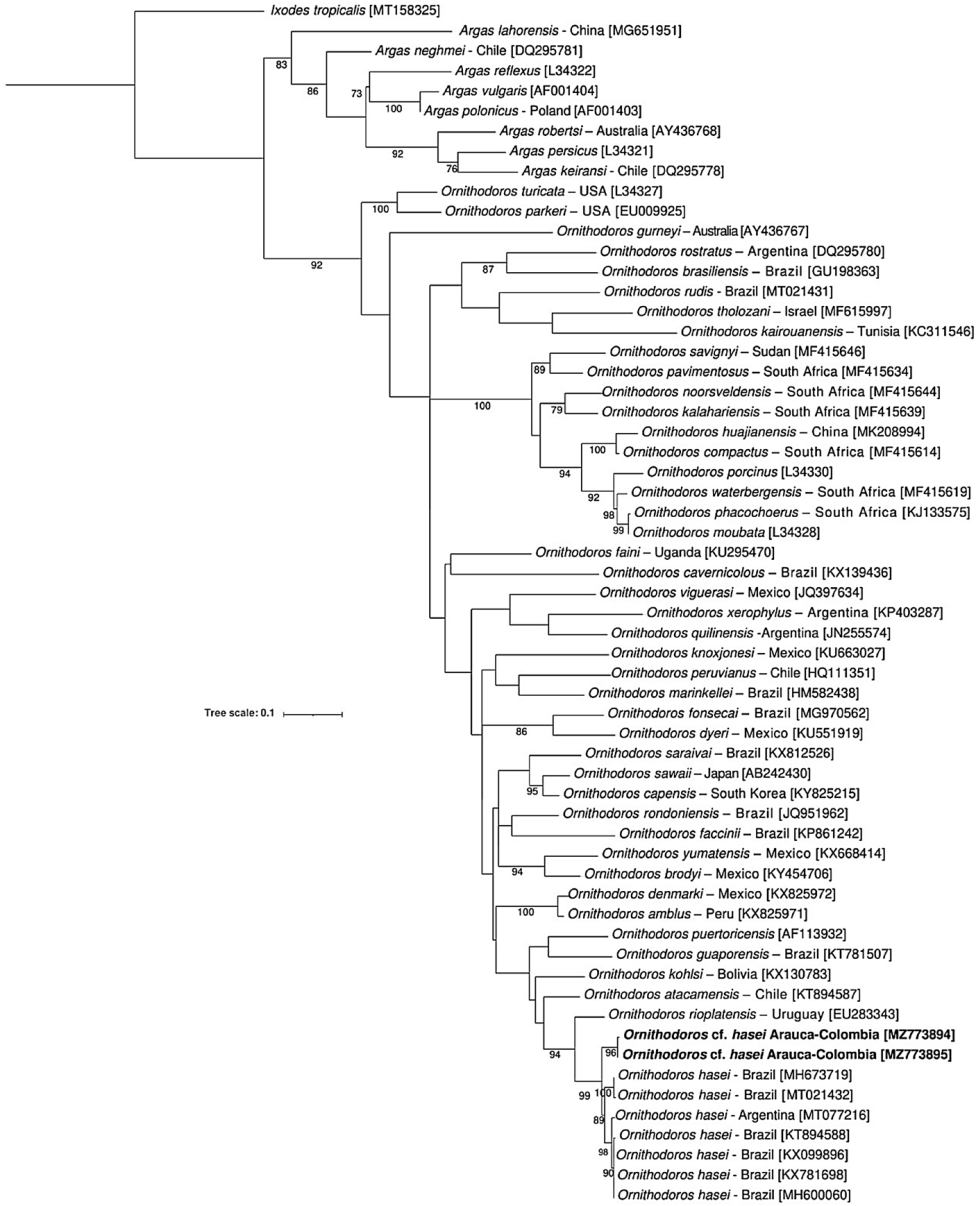
Phylogenetic tree inferred by maximum likelihood (ML) by partial gene alignment of 16SrDNA. The positions of *Ornithodoros* cf. *hasei* identified in the study are indicated in bold. ML Bootstrap are indicated in each of the clades respectively. GenBank hits are shown in brackets

In total, 169 soft ticks of the genus *Ornithodoros* were collected from 19 bats: 163 larvae and 6 nymphs (Table S1). The ticks involved in the study were morphologically and molecularly assigned to *O.* cf. *hasei*, based on the following combination of traits: 19 pairs of setae on the dorsal, three pair sternal setae (ventral), three pair circumanal setae (ventral), four central setae (dorsal), seven pairs of anterolateral setae (dorsal), eight pairs of posterolateral setae (dorsal) and pointed end hypostome with three rows of teeth on the distal end. The 16 S rDNA gene sequences showed a similarity of 95.3–95.6% with other sequences belonging to *O. hasei.* Nymphs were only confirmed molecularly as *O.* cf. *hasei* comparing the sequences with the larvae (100% similarity). ML analysis placed this species with other *O. hasei* specimens from Brazil and Argentina (Fig. 1).

**Fig. 2:**
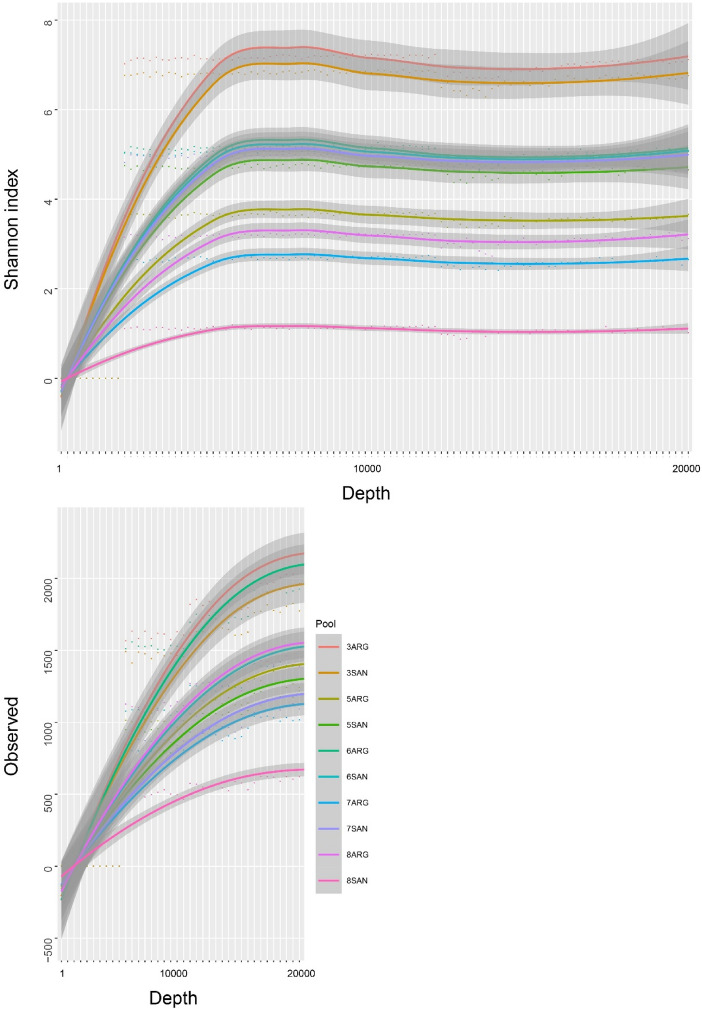
Shannon index and species accumulation curves (Observed) for each pool sequenced

**Fig. 3:**
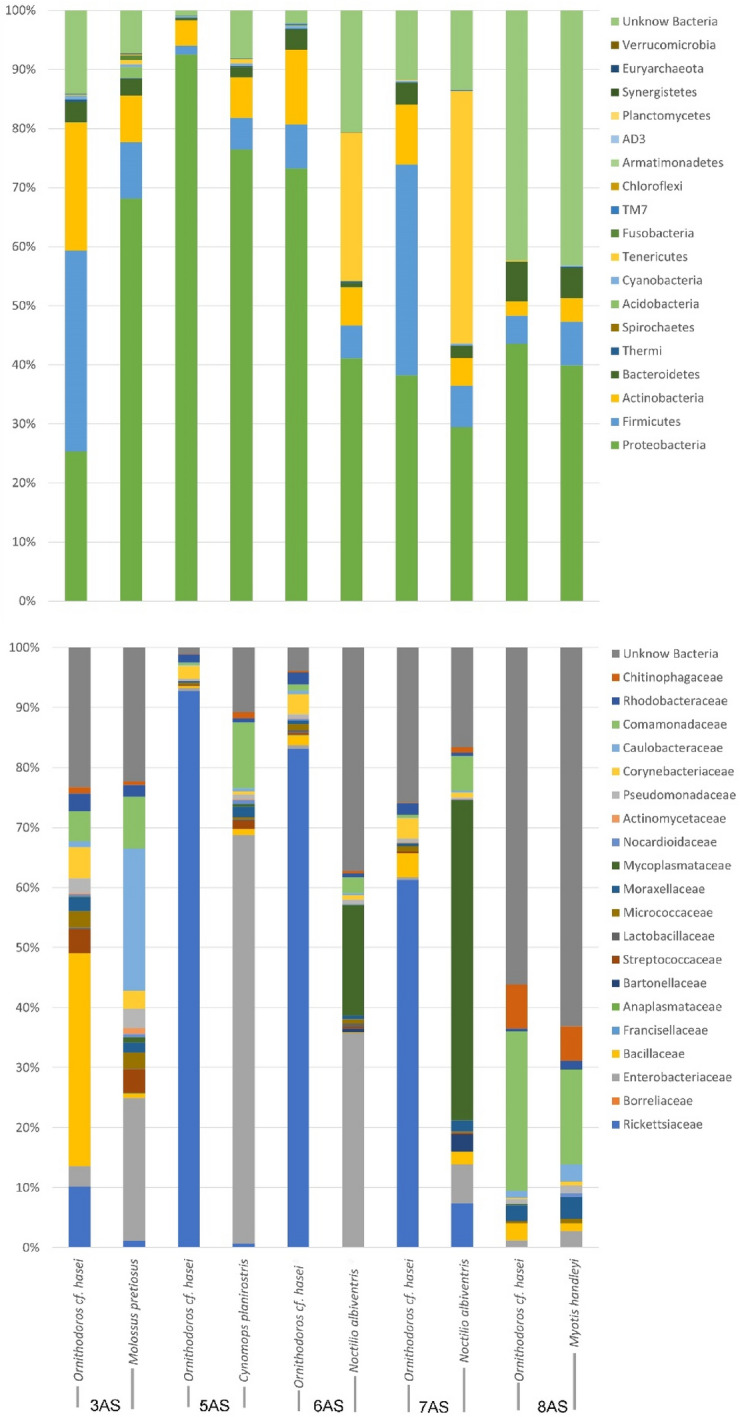
Relative abundance of bacterial taxa in *Ornithodoros* cf. *hasei* from the blood of their mammalian hosts. Bacterial taxa are grouped at the phyla (top panel) and family (bottom panel) level. Every bar represents a pooled sample for *O.* cf. *hasei* and the corresponding mammalian host blood (Table 1). The order of taxa and their respective color is consistent from top to bottom

In total 3,427924 sequencing reads were obtained from the total samples; 1,613538 reads from tick samples and 1,814386 reads from mammalian blood samples. The average number of reads for each sample was 107123 (57288–154859) and with an average length of 297 bp. The Shannon index and the species accumulation curves showed that all samples except for 8SAN-pool manage to achieve a correct species richness upon sampling (Fig. 2). Argasid pools presented a higher diversity (higher number of taxa described) of bacteria found, as well as a higher average number of reads (115252) compared to host blood (100799) (Table S2). Based on the analysis of bacterial abundances and identification, bacterial groups belonging to Proteobacteria (38.8%), Enterobacteriaceae (25%), Firmicutes (12.3%) and Actinobacteria (10.9%) were found in mammalian blood. For ticks, higher abundances were found for Rickettsiaceae (39%), Firmicutes (25%), Actinobacteria (13.1%) and Proteobacteria (9%) and between 0.8 and 1.41% was not taxonomically assigned (Fig. 3).

**Fig. 4:**
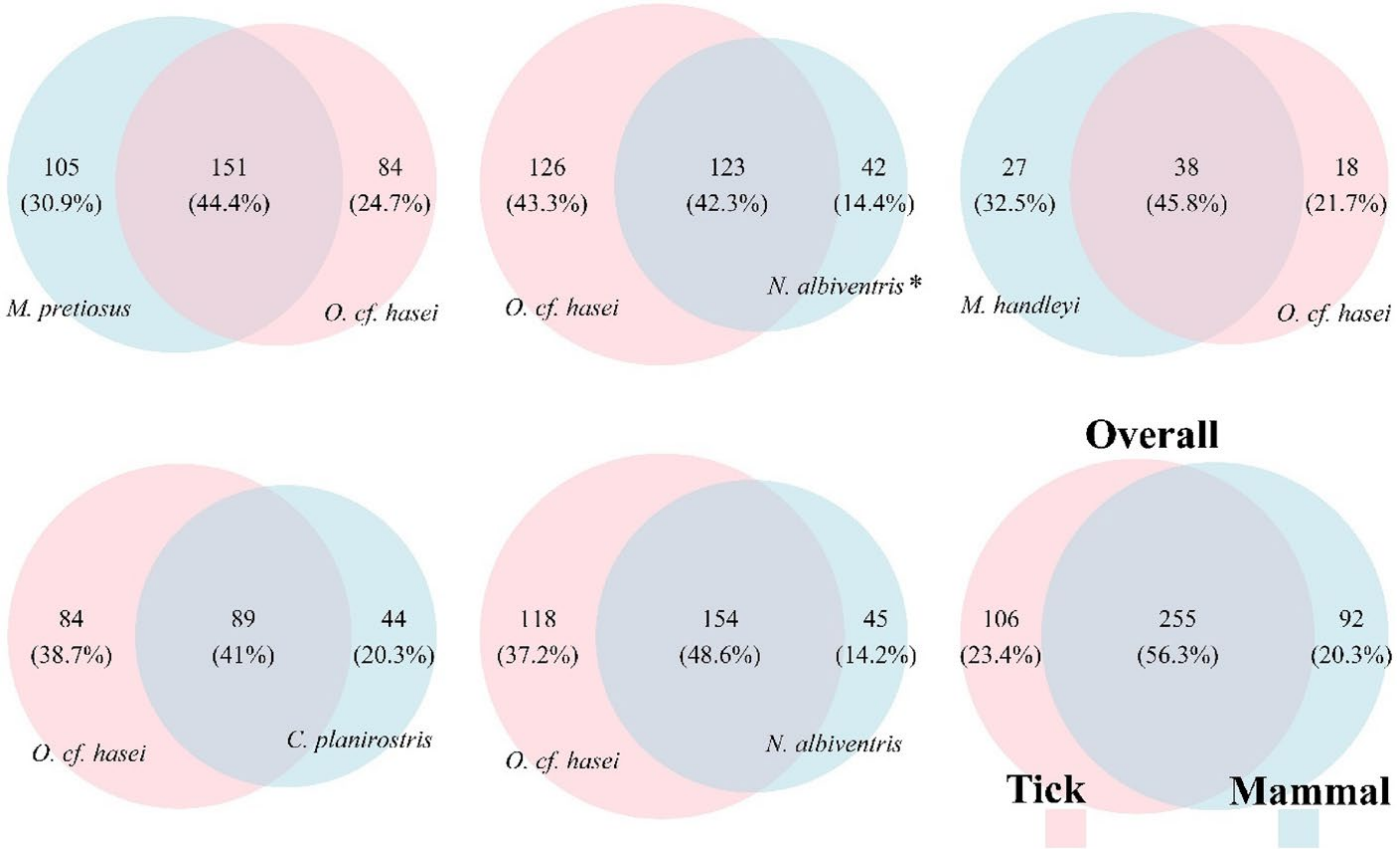
Operational taxonomic units (OTUs) shared at the genus and species level for each tick pool and corresponding mammalian host blood. The overall represent all the genus and species OTUs found in the whole pools of tick and mammals

The readings confirmed the presence of tick-associated pathogenic bacteria, which were found on the families Anaplasmacetaceae, Bartonellaceae, Borreliaceae, Francisellaceae and Rickettsiaceae (Table 2). Rickettsiaceae presented the highest abundance (up to 85%), represented mainly by *Rickettsia*-like endosymbionts. The identification of the deepest taxonomic level for the pathogenic species resulted as *Borrelia* sp., *Bartonella tamiae*, *Ehrlichia* sp., *Francisella cantonensis*, and *Rickettsia-*like endosimbiont. *Bartonella tamiae* and *Francisella cantonensis* species were not found to be present in both tick and host blood samples. *Francisella cantonensis* was only detected in *O.* cf. *hasei.* Analysis of shared OTUs (genera and species) between ticks and host blood showed that about 41–48.6% is shared (Fig. 4).

**Table 2:** Description of taxa detected in the study related to ticks, abundance, and their relationship with hosts (direct; present in ticks and host and not direct; present in only one organism)

Host	Relation	Family	Genus	Abundance (%)
*O.* cf. *hasei – M. pretiosus*	Direct (*M. pretiosus – O.* cf. *hasei*)	Anaplasmacetaceae	*Ehrlichia* sp.	0.005–0.091
*O.* cf. *hasei*, *N. albiventris* and *C. planirostris*	Direct (*N. albiventris – O.* cf. *hasei*)	Borreliaceae	*Borrelia* sp.	0.007–2.61
*O.* cf. *hasei – N. albiventris*	Not direct	Bartonellaceae	*Bartonella tamiae*	0.008–5.26
*O.* cf. *hasei*	Not direct	Francisellaceae	*Francisella cantonensis*	0.01
*O.* cf. *hasei*, *N. albiventris*, *C. planirostris* and *M. pretiosus*	Direct (*N. albiventris/M. pretiosus/C. planirostris – O.* cf. *hasei*)	Rickettsiaceae	*Rickettsia*-like endosimbiont	0.08–85

## Discussion

Our results demonstrate the high diversity of bacteria found in both the blood of small wild mammals and ticks. The results agree with previous studies where Actinobacteria, Proteobacteria and Firmicutes, are the most common bacteria in the tick-related microbiome (Narasimhan and Fikrig 2015), such as *O. turicata* (Barraza-Guerrero et al. 2020), *A.* *americanum* (Maldonado-Ruiz et al. 2021) and *A.* *tuberculatum* (Budachetri et al. 2016). Moreover, Proteobacteria has been related as one of the most common and dominant bacterial types in the tick microbiome in general, and it has been reported as the main abundant taxon in species such as *A. japonicus* (Yan et al. 2019), *Dermacentor marginatus*, *I. ricinus*, and *Rhiphicephalus sanguineus* (Portillo et al. 2019). On the other hand, although Proteobacteria is abundant in the blood of mammals, in ticks it was the most prominent in only half of the pools, Rickettsiaceae dominating the other half. The dominance of Rickettsiaceae is comprised on two taxa: *Rickettsia*-like endosymbionts and *Wolbachia*-like endosymbionts (Table 2). These two taxa have been considered as parasites of ticks and in the case of *Rickettsia* as disease-causing in vertebrates (Miranda et al. 2012; Plantard et al. 2012; Rivera-Páez et al. 2018a, b; López-Pérez et al. 2019; Muñoz-Leal et al. 2019; Bobo 2020), and specifically related to soft ticks (Duh et al. 2010; Sánchez-Montes et al. 2016; Muñoz-Leal et al. 2019; Han et al. 2021; Peixoto et al. 2021). In addition, both have been related to the transmission or interference to other pathogens or bacterial communities (Haine 2008; Walker et al. 2011).

Other tick-related pathogenic bacteria include the families Anaplasmacetaceae, Bartonellaceae, Borreliaceae and Francisellaceae (Table 1). In the case of Anaplasmacetaceae, *Ehrlichia* has been reported widely for ticks of the genus *Amblyomma* and *Rhiphicephalus* (Bekker et al. 2002; Loftis et al. 2006; Stich et al. 2008; Doudier et al. 2010). Although *Ehrlichia* is not related with soft ticks, it has been listed as an effective pathogen in circulation among mammals, such as *E. canis* (Stich et al. 2008). Therefore, central studies in mammals could reveal much more information about its cycle and related vectors. *Ehrlichia* sp. was detected only in ticks and in the blood one bat host (*Molossus pretiosus*) (Table 2). Meanwhile, *Bartonella* and *Francisella* have been most frequently associated with tick-borne transmission, which may contribute to disease in humans (Sun et al. 2000; Chang et al. 2001; Johnson et al. 2003; Scoles 2004; Chomel et al. 2006; Petersen et al. 2009; Gerhart et al. 2016; Wechtaisong et al. 2020). Specifically, *Bartonella tamiae* has been considered a notable pathogen due to reports of blood in humans in Thailand (Kosoy et al. 2008), and ticks of the genus *Ixodes* in Algeria (Leulmi et al. 2016). *Francisella cantonensis* is known primarily as an aquatic species (Duodu et al. 2012), though it has been isolated from air systems (Qu et al. 2009). The ability of *F. cantonensis* to act as a pathogen has yet to be determined in humans or other animals, though this requires more research (Qu et al. 2009; Duodu et al. 2012). Given this, the detection of the genus in metagenomic methodologies is critical, as the identification of species-level using a short 16 S segment is highly variable (Poretsky et al. 2014).

*Borrelia* was primarily detected in wild mammal species in the study (*C. planirostris*, *N. albiventris*). Despite this prevalence, OTUs analysis did not determine a preferred species for *Borrelia* readings, which could suggest the presence of a new species. Reports of *Borrelia* in ticks and wild mammals have been increasing in recent years in South America (Ataliba et al. 2007; Kelly et al. 2014; Muñoz-Leal et al. 2018, 2021b; Morel et al. 2019; Sánchez et al. 2020), but with few records in Colombia (Marinkelle and Grose 1968; Muñoz-Leal et al. 2021b). *Borrelia* and *Rickettsia* have been the only confirmed potential pathogenic bacteria related to *O. hasei* (Tahir et al. 2016; Colombo et al. 2020; Muñoz-Leal et al. 2021a), however, all recorded pathogenic genera were detected in *O*. cf. *hasei* (Table 2), which relates this tick to the presence of other pathogenic bacteria that have not been studied or classified. The presence of these bacteria could come directly from contamination of blood shared by hosts (Wu-Chuang et al. 2021). Therefore, the role of *O. hasei* and the maintenance of pathogenic bacteria from hosts has not been fully studied, and the role of *O. hasei* and its relationship to the presence of *Borrelia* in South America is still under investigation (Shapiro and Gerber 2011; Wang 2015; Robles et al. 2018; O’Keeffe et al. 2020).

Several studies have been conducted on the shared bacterial assemblages in the microbiome of ticks and blood of their wild hosts (Zhang et al. 2014; Swei and Kwan 2017), as well as in experimental methodologies (Rynkiewicz et al. 2015). These studies have identified a large variety of bacterial species that could be potential pathogens for vertebrates, and a large percentage of bacterial communities that could be transmitted during the feeding process (Zhang et al. 2014; Rynkiewicz et al. 2015; Swei and Kwan 2017). The analysis of OTUs in our study showed that about 41–48.6% are shared between ticks and their hosts (Fig. 4). Previous studies have shown that blood from wild hosts and the suction process during tick feeding is a possible mechanism for sharing of bacterial assemblages (Rynkiewicz et al. 2015; Swei and Kwan 2017). Moreover, there are several factors that may involve host-parasite contamination, such as the presence of non-pathogenic symbiotic communities from the surrounding environment of the sample and errors in sample handling (Wu-Chuang et al. 2021). Thus, the results of this study may suggest that, although a large number of OTUs are shared, only a small proportion may actually be transmissible, and a smaller proportion has pathogenic potential. Other studies have related the shared microbiome to non-pathogenic symbiont species, though this may be confounded by low resolution in species identification analyses (Rynkiewicz et al. 2015; Barraza-Guerrero et al. 2020; Wu-Chuang et al. 2021).

## Conclusions

The detection of genera such as *Ehrlichia*, *Bartonella*, *Borrelia* and *Rickettsia*, allows the involvement of a significant and direct relationship between ticks and wild mammals. However, transmission by other related vectors in the study area must be elucidated. Wild mammals are rarely included in microbiome screening studies or in the search for new bacteria with pathogenic potential. In this sense, the inclusion of these species is emphasized in order to elucidate the dynamics of the disease ecology in which they may be involved.

## Supplementary Information

Below is the link to the electronic supplementary material.

Supplementary material 1 (DOCX 18 kb)

Supplementary material 2 (XLSX 13 kb)
Supplementary information about statistics of sequence data (reads) and numbers757 of taxa identified of each pool

## References

[CR1] Alkayyoomi RN (2018) Metagenomic profile of the bacterial communities associated with *Ornithodoros Muesebecki* (Acari: Argasidae) ticks on Socotra cormorant colony in the United Arab Emirates and presence of three important pathogenic groups in them. United Arab Emirates University. Biology thesis

[CR2] Andreotti R, de Leon AAP, Dowd SE, Guerrero FD, Bendele KG, Scoles GA (2011). Assessment of bacterial diversity in the cattle tick *Rhipicephalus (Boophilus) microplus* through tag-encoded pyrosequencing. BMC Microbiol.

[CR3] Ataliba AC, Resende JS, Yoshinari N, Labruna MB (2007). Isolation and molecular characterization of a Brazilian strain of *Borrelia anserina*, the agent of fowl spirochaetosis. Res Vet Sci.

[CR4] Barraza-Guerrero SI, Meza-Herrera CA, García-De la Peña C, González-Álvarez VH, Vaca-Paniagua F, Díaz-Velásquez CE, Sánchez-Tortosa F, Ávila-Rodríguez V, Valenzuela-Núñez LM, Herrera-Salazar JC (2020). General microbiota of the soft tick *Ornithodoros turicata* parasitizing the *Bolson Tortoise* (Gopherus flavomarginatus) in the Mapimi biosphere reserve. Mexico Biol.

[CR5] Battesti DMB, Arzua M, Bechara GH (2006) Carrapatos de importância médico-veterinária da região neotropical: um guia ilustrado para identificação de espécies, in: Carrapatos de Importância Médico-Veterinária Da Região Neotropical: Um Guia Ilustrado Para Identificação de Espécies. pp. xvi–223

[CR6] Bazán-León EA (2011) Ecología parasitaria de dos especies de pequeños mamíferos de Chile, *Abrothrix olivaceus* (Rodentia-Cricetidae) y *Thylamys elegans* (Didelphimorphia-Didelphidae), *Abrothrix olivaceus* (Rodentia: Cricetidae) y *Thylamys elegans* (Didelphimorphia: Didelphidae). Tesis (Magíster en Ciencias, mención Ecología y Biología Evolutiva). Santiago, Chile. Universidad de Chile, Facultad de Ciencias, 2011. 108 p

[CR7] Bekker CPJ, De Vos S, Taoufik A, Sparagano OAE, Jongejan F (2002). Simultaneous detection of *Anaplasma* and *Ehrlichia* species in ruminants and detection of *Ehrlichia ruminantium* in *Amblyomma variegatum* ticks by reverse line blot hybridization. Vet Microbiol.

[CR8] Bennett S (2017) The complex eco-epidemiology of tick borne disease: ticks, hosts and pathobiomes in an urbanizing environment. University of Minnesota Ph.D. dissertation. https://hdl.handle.net/11299/191397

[CR9] Binetruy F, Dupraz M, Buysse M, Duron O (2019). Surface sterilization methods impact measures of internal microbial diversity in ticks. Parasites & Vectors.

[CR10] Bobo CG (2020) Molecular characterization of *Wolbachia* and its impact on the microbiome of exotic and united states ticks. The University of Southern Mississippi. Honors Bachelor Theses. 702. https://aquila.usm.edu/honors_theses/702

[CR11] Bonnet SI, Binetruy F, Hernandez-Jarguin AM, Duron O (2017). The tick microbiome: why non-pathogenic microorganisms matter in tick biology and pathogen transmission. Front Cell Infect Microbiol.

[CR12] Budachetri K, Browning RE, Adamson SW, Dowd SE, Chao C-C, Ching W-M, Karim S (2014). An insight into the microbiome of the *Amblyomma maculatum* (Acari: Ixodidae). J Med Entomol.

[CR13] Budachetri K, Gaillard D, Williams J, Mukherjee N, Karim S (2016). A snapshot of the microbiome of *Amblyomma tuberculatum* ticks infesting the gopher tortoise, an endangered species. Ticks Tick Borne Dis.

[CR14] Cabezas-Cruz A, Vayssier-Taussat M, Greub G (2018). Tick-borne pathogen detection: what’s new?. Microbes Infect.

[CR15] Camicas JL, Hervy JP, Adam F, Morel PC (1998) The ticks of the world (Acarida, Ixodida): nomenclature, described stages, hosts, distribution. Éditions de l’Orstom, Paris. The Food and Agriculture Organization (FAO)

[CR16] Carpi G, Cagnacci F, Wittekindt NE, Zhao F, Qi J, Tomsho LP, Drautz DI, Rizzoli A, Schuster SC (2011). Metagenomic profile of the bacterial communities associated with *Ixodes ricinus* ticks. PLoS ONE.

[CR17] Chang CC, Chomel BB, Kasten RW, Romano V, Tietze N (2001). Molecular evidence of Bartonella spp. in questing adult Ixodes pacificus ticks in California. J Clin Microbiol.

[CR18] Chomel BB, Boulouis H-J, Maruyama S, Breitschwerdt EB (2006). *Bartonella* spp. in pets and effect on human health. Emerg Infect Dis.

[CR19] Colombo VC, Montani ME, Pavé R, Antoniazzi LR, Gamboa MD, Fasano AA, Félix ML, Nava S, Venzal JM (2020). First detection of “*Candidatus* Rickettsia wissemanii” in *Ornithodoros hasei* (Schulze,1935) (Acari: Argasidae) from Argentina. Ticks Tick Borne Dis.

[CR20] Dantas-Torres F, Fernandes Martins T, Muñoz-Leal S, Onofrio VC, Barros-Battesti DM (2019). Ticks (Ixodida: Argasidae, Ixodidae) of Brazil: updated species checklist and taxonomic keys. Ticks Tick Borne Dis.

[CR21] de Oliveira GMB, da Silva IWG, da Cruz Ferreira Evaristo AM, de Azevedo Serpa MC, Silva Campos AN, Dutra V, Nakazato L, de Aguiar DM, Bahia Labruna M, Horta MC (2020). Tick-borne pathogens in dogs, wild small mammals and their ectoparasites in the semi-arid Caatinga biome, northeastern Brazil. Ticks Tick Borne Dis.

[CR22] Diaz A (2021). Flaviviruses and where the Zika virus fits in: an overview. Zika Virus Biol Transm Pathol.

[CR23] Droop AP (2016). fqtools: an efficient software suite for modern FASTQ file manipulation. Bioinformatics.

[CR24] Doudier B, Olano J, Parola P, Brouqui P (2010). Factors contributing to emergence of *Ehrlichia* and *Anaplasma* spp. as human pathogens. Veterinary Parasitol.

[CR25] Duh D, Punda-Polic V, Avsic-Zupanc T, Bouyer D, Walker DH, Popov VL, Jelovsek M, Gracner M, Trilar T, Bradaric N, Kurtti TJ, Strus J (2010). *Rickettsia hoogstraalii* sp. nov., isolated from hardand soft-bodied ticks. Int J Syst Evol Microbiol.

[CR26] Duodu S, Larsson P, Sjödin A, Forsman M, Colquhoun DJ (2012). The distribution of Francisella-like bacteria associated with coastal waters in Norway. Microb Ecol.

[CR27] Esser HJ, Herre EA, Blüthgen N, Loaiza JR, Bermúdez SE, Jansen PA (2016). Host specificity in a diverse neotropical tick community: an assessment using quantitative network analysis and host phylogeny. Parasit Vectors.

[CR28] Estaki M, Jiang L, Bokulich NA, McDonald D, González A, Kosciolek T, Martino C, Zhu Q, Birmingham A, Vázquez-Baeza Y (2020). QIIME 2 enables comprehensive end‐to‐end analysis of diverse microbiome data and comparative studies with publicly available data. Curr Protoc Bioinforma.

[CR29] Estrada-Peña A, Gray JS, Kahl O, Lane RS, Nijhoff AM (2013). Research on the ecology of ticks and tick-borne pathogens—methodological principles and caveats. Front Cell Infect Microbiol.

[CR30] Faccini-Martínez ÁA, Botero-García CA (2016). Regarding tick-borne relapsing fever in the Americas: some historical aspects of a forgotten disease in Colombia. Vet Sci.

[CR31] Faccini-Martínez ÁA, Ramírez-Hernández A, Forero-Becerra E, Cortés-Vecino JA, Escandón P, Rodas JD, Palomar AM, Portillo A, Oteo JA, Hidalgo M (2016). Molecular evidence of different *Rickettsia* Species in Villeta, Colombia. Vector-Borne Zoonotic Dis.

[CR32] Fadrosh DW, Ma B, Gajer P, Sengamalay N, Ott S, Brotman RM, Ravel J (2014). An improved dual-indexing approach for multiplexed 16S rRNA gene sequencing on the Illumina MiSeq platform. Microbiome.

[CR33] Filippova N (1966). Argasid ticks (Argasidae). Fauna SSSR Paukoobraznye.

[CR34] Franco R, Toro G, Martinez J (1911). Fiebre amarilla y fiebre espiroquetal. Ses. Científicas del Centen. Acad Nac Med Bogota.

[CR35] Gardner AL (2008). Mammals of South America, volume 1: marsupials, xenarthrans, shrews, and bats.

[CR36] Gerhart JG, Moses AS, Raghavan R (2016). A Francisella-like endosymbiont in the gulf coast tick evolved from a mammalian pathogen. Sci Rep.

[CR37] Greay TL, Gofton AW, Paparini A, Ryan UM, Oskam CL, Irwin PJ (2018). Recent insights into the tick microbiome gained through next-generation sequencing. Parasit Vectors.

[CR38] Guglielmone AA (2021). Neotropical hard ticks (Acari: Ixodida: Ixodidae): a critical analysis of their taxonomy, distribution, and host relationships.

[CR39] Haine ER (2008). Symbiont-mediated protection. Proc R Soc B Biol Sci.

[CR40] Han S-W, Chae J-B, Jo Y-S, Cho Y-K, Kang J-G, Shin N-S, Youn H-J, Youn H-Y, Nam H-M, Kim H-J (2021). First detection of *Borrelia* and *Rickettsia* species from *Ornithodoros* ticks in the Republic of Korea. Ticks Tick Borne Dis.

[CR41] Hanafi-Bojd AA, Jafari S, Telmadarraiy Z, Abbasi-Ghahramanloo A, Moradi-Asl E (2021). Spatial distribution of ticks (Arachniada: Argasidae and Ixodidae) and their infection rate to crimean-Congo hemorrhagic fever virus in Iran. J Arthropod Borne Dis.

[CR42] Hidalgo M, Miranda J, Heredia D, Zambrano P, Vesga JF, Lizarazo D, Mattar S, Valbuena G (2011). Outbreak of rocky mountain spotted fever in Córdoba. Colombia Mem Inst Oswaldo Cruz.

[CR43] Hoogstraal H (1985). Argasid and nuttalliellid ticks as parasites and vectors. Adv Parasitol.

[CR44] Huson DH, Beier S, Flade I, Górska A, El-Hadidi M, Mitra S, Tappu R (2016). MEGAN community edition-interactive exploration and analysis of large-scale microbiome sequencing data. PLoS Comput Biol.

[CR300] Huson DH, Mitra S (2012). Introduction to the analysis of environmental sequences: metagenomics with MEGAN. Evolutionary Genomics.

[CR45] Johnson G, Ayers M, McClure SCC, Richardson SE, Tellier R (2003). Detection and identification of *Bartonella* species pathogenic for humans by PCR amplification targeting the riboflavin synthase gene (ribC). J Clin Microbiol.

[CR46] Kaehler BD, Bokulich NA, McDonald D, Knight R, Caporaso JG, Huttley GA (2019). Species abundance information improves sequence taxonomy classification accuracy. Nat Commun.

[CR47] Kazim AR, Houssaini J, Ehlers J, Tappe D, Heo CC (2021). Soft ticks (Acari: Argasidae) in the island nations of Southeast Asia: a review on their distribution, associated hosts and potential pathogens. Acta Trop.

[CR48] Kelly AL, Raffel SJ, Fischer R, Bellinghausen M, Stevenson C, Schwan TG (2014). First isolation of the relapsing fever spirochete, *Borrelia herrnsii*, from a domestic dog. Ticks Tick Borne Dis.

[CR49] Klompen JSH, Oliver JH (1993). Systematic relationships in the soft ticks (Acari: Ixodida: Argasidae). Syst Entomol.

[CR50] Kosoy M, Morway C, Sheff KW, Bai Y, Colborn J, Chalcraft L, Dowell SF, Peruski LF, Maloney SA, Baggett H (2008). *Bartonella tamiae* sp. nov., a newly recognized pathogen isolated from three human patients from Thailand. J Clin Microbiol.

[CR51] Letunic I, Bork P (2019). Interactive tree of life (iTOL) v4: recent updates and new developments. Nucleic Acids Res.

[CR52] Leulmi H, Aouadi A, Bitam I, Bessas A, Benakhla A, Raoult D, Parola P (2016). Detection of *Bartonella tamiae*, *Coxiella burnetii* and rickettsiae in arthropods and tissues from wild and domestic animals in northeastern Algeria. Parasit Vectors.

[CR53] Loftis AD, Gill JS, Schriefer ME, Levin ML, Eremeeva ME, Gilchrist MJR, Dasch GA (2005). Detection of *Rickettsia*, *Borrelia*, and *Bartonella* in Carios kelleyi (Acari: Argasidae). J Med Entomol.

[CR54] Loftis AD, Reeves WK, Spurlock JP, Mahan SM, Troughton DR, Dasch GA, Levin ML (2006). Infection of a goat with a tick-transmitted *Ehrlichia* from Georgia, USA, that is closely related to *Ehrlichia ruminantium*. J Vector Ecol.

[CR55] López-Pérez AM, Sánchez-Montes S, Foley J, Guzmán-Cornejo C, Colunga-Salas P, Pascoe E, Becker I, Delgado-de la Mora J, Licona-Enriquez JD, Suzan G (2019). Molecular evidence of *Borrelia burgdorferi* sensu stricto and *Rickettsia massiliae* in ticks collected from a domestic-wild carnivore interface in Chihuahua, Mexico. Ticks Tick Borne Dis.

[CR56] López Y, Robayo-Sánchez LN, Muñoz-Leal S, Aleman A, Arroyave E, Ramírez-Hernández A, Cortés-Vecino JA, Mattar S, Faccini-Martínez ÁA (2021). *Ornithodoros puertoricensis* (Ixodida: Argasidae) associated with domestic fowl in rural dwellings from Córdoba department, Caribbean Colombia. Front Vet Sci.

[CR57] Luz HR, Muñoz-Leal S, de Carvalho WD, Castro IJ, Xavier BS, Toledo JJ, Hilário R, Acosta ICLL, Faccini JLH, Labruna MB (2019). Detection of “*Candidatus* Rickettsia wissemanii” in ticks parasitizing bats (Mammalia: Chiroptera) in the northern Brazilian Amazon. Parasitol Res.

[CR58] Magoč T, Salzberg SL (2011). FLASH: fast length adjustment of short reads to improve genome assemblies. Bioinformatics.

[CR59] Maldonado-Ruiz LP, Neupane S, Park Y, Zurek L (2021). The bacterial community of the lone star tick (*Amblyomma americanum*). Parasit Vectors.

[CR60] Mangold AJ, Bargues MD, Mas-Coma S (1998). Mitochondrial 16S rDNA sequences and phylogenetic relationships of species of *Rhipicephalus* and other tick genera among Metastriata (Acari: Ixodidae). Parasitol Res.

[CR61] Manzano-Román R, Díaz-Martín V, de la Fuente J, Pérez-Sánchez R (2012). Soft ticks as pathogen vectors: distribution, surveillance and control. Parasitology.

[CR62] Marinkelle CJ, Grose ES (1968). Species of borrelia from a Colombian bat (*Natalus tumidirostris*). Nature.

[CR63] Marinkelle CJ, Grose ES (1981). A list of ectoparasites of Colombian bats. Rev Biol Trop.

[CR64] Martin M (2011). Cutadapt removes adapter sequences from high-throughput sequencing reads. EMBnet J.

[CR65] Menchaca AC, Visi DK, Strey OF, Teel PD, Kalinowski K, Allen MS, Williamson PC (2013). Preliminary assessment of microbiome changes following blood-feeding and survivorship in the *Amblyomma americanum* nymph-to-adult transition using semiconductor sequencing. PLoS ONE.

[CR66] Miller MA, Pfeiffer W, Schwartz T (2011) The CIPRES science gateway: a community resource for phylogenetic analyses. In: Proceedings of the 2011 TeraGrid conference: extreme digital discovery (pp. 1–8).10.1016/B978-1-4160-6400-8.00017-1

[CR67] Miranda J, Portillo A, Oteo JA, Mattar S (2012). Rickettsia sp. strain colombianensi (Rickettsiales: Rickettsiaceae): A new proposed rickettsia detected in *Amblyomma dissimile* (Acari: Ixodidae) from iguanas and free-living larvae ticks from vegetation. J Med Entomol.

[CR68] Morel N, De Salvo MN, Cicuttin G, Rossner V, Thompson CS, Mangold AJ, Nava S (2019). The presence of *Borrelia theileri* in Argentina. Vet Parasitol Reg Stud Rep.

[CR69] Muñoz-Leal S, Faccini-Martinez AA, Costa FB, Marcili A, Mesquita ETKC, Marques EP, Labruna MB (2018). Isolation and molecular characterization of a relapsing fever Borrelia recovered from *Ornithodoros rudis* in Brazil. Ticks Tick Borne Dis.

[CR70] Muñoz-Leal S, Macedo C, Goncalves TC, Barreira JD, Labruna MB, Sampaio de Lemos ER, Ogrzewalska M (2019). Detected microorganisms and new geographic records of *Ornithodoros rietcorreai* (Acari: Argasidae) from northern Brazil. Ticks Tick Borne Dis.

[CR71] Muñoz-Leal S, Faccini-Martínez ÁA, Teixeira BM, Martins MM, Serpa MCA, Oliveira GMB, Jorge FR, Pacheco RC, Costa FB, Luz HR (2021). Relapsing fever group borreliae in human-biting soft ticks, Brazil. Emerg Infect Dis.

[CR72] Muñoz-Leal S, Faccini‐Martínez ÁA, Pérez‐Torres J, Chala‐Quintero SM, Herrera‐Sepúlveda MT, Cuervo C, Labruna MB (2021). Novel *Borrelia* genotypes in bats from the Macaregua Cave, Colombia. Zoonoses Public Health.

[CR73] Narasimhan S, Fikrig E (2015). Tick microbiome: the force within. TRENDS Parasitol.

[CR74] Nava S, Venzal JMM, González-Acuña DG, Martins TFF, Guglielmone AAA (2017). Ticks of the southern cone of America: diagnosis, distribution, and hosts with taxonomy, ecology and sanitary importance.

[CR75] O’Keeffe KR, Oppler ZJ, Brisson D (2020). Evolutionary ecology of lyme borrelia. Infect Genet Evol.

[CR76] Oksanen J, Blanchet FG, Kindt R, Legendre P, Minchin PR, O’hara RB, Simpson GL, Solymos P, Stevens MHH, Wagner H (2013) Package ‘vegan.’ Community Ecol. Packag. version 2, 1–295. http://CRAN.Rproject.org/package=vegan

[CR77] Ortíz-Giraldo M, Tobón-Escobar WD, Velásquez-Guarín D, Usma-Marín MF, Ossa-López PA, Ramírez-Chaves HE, Carvajal-Agudelo JD, Rivera-Páez FA (2021). Ticks (Acari: Ixodoidea) associated with mammals in Colombia: a historical review, molecular species confirmation, and establishment of new relationships. Parasitol Res.

[CR78] Peixoto MP, Luz HR, de Abreu DPB, Faccini JLH, McIntosh D (2021). Detection of *Rickettsia* sp. strain Itinguçú in *Ornithodoros faccinii* (Acari: Argasidae) parasitizing the toad *Rhinella ornata* (Anura: Bufonidae) in Brazil. Ticks Tick Borne Dis.

[CR79] Petersen JM, Mead PS, Schriefer ME (2009). *Francisella tularensis*: an arthropod-borne pathogen. Vet Res.

[CR80] Pino Pou R (1984) La fiebre recurrente en general y particularmente en Venezuela, in: Trabajos Cientificos y Discursos de Incorporación a La Academia Nacional de Medicina 1915–1923; Tomo II. pp. 143–221

[CR81] Plantard O, Bouju-Albert A, Malard M-A, Hermouet A, Capron G, Verheyden H (2012). Detection of *Wolbachia* in the tick *Ixodes ricinus* is due to the presence of the hymenoptera endoparasitoid *Ixodiphagus hookeri*. PLoS ONE.

[CR82] Poretsky R, Rodriguez-R LM, Luo C, Tsementzi D, Konstantinidis KT (2014). Strengths and limitations of 16S rRNA gene amplicon sequencing in revealing temporal microbial community dynamics. PLoS ONE.

[CR83] Portillo A, Palomar AM, de Toro M, Santibáñez S, Santibáñez P, Oteo JA (2019). Exploring the bacteriome in anthropophilic ticks: to investigate the vectors for diagnosis. PLoS ONE.

[CR84] Qu P, Deng X, Zhang J, Chen J, Zhang Q, Xiao Y, Chen S (2009). Identification and characterization of the *Francisella* sp. strain 08HL01032 isolated in air condition systems. Acta Microbiol Sin.

[CR85] Rivera-Páez FA, Labruna MB, Martins TF, Perez JE, Castaño-Villa GJ, Ossa-López PA, Gil CA, Sampieri BR, Aricapa-Giraldo HJ, Camargo-Mathias MI (2018). Contributions to the knowledge of hard ticks (Acari: Ixodidae) in Colombia. Ticks Tick Borne Dis.

[CR86] Rivera-Páez FA, Martins TF, Ossa-López PA, Sampieri BR, Camargo-Mathias MI (2018). Detection of *Rickettsia* spp. in ticks (Acari: Ixodidae) of domestic animals in Colombia. Ticks Tick Borne Dis.

[CR87] Robles A, Fong J, Cervantes J (2018). Borrelia infection in latin America. Rev Investig Clín.

[CR88] Rognes T, Flouri T, Nichols B, Quince C, Mahé F (2016). VSEARCH: a versatile open source tool for metagenomics. PeerJ.

[CR89] Ronquist F, Teslenko M, Van Der Mark P, Ayres DL, Darling A, Höhna S, Huelsenbeck JP (2012). MrBayes 3.2: efficient Bayesian phylogenetic inference and model choice across a large model space. Syst Biol.

[CR90] Rynkiewicz EC, Hemmerich C, Rusch DB, Fuqua C, Clay K (2015). Concordance of bacterial communities of two tick species and blood of their shared rodent host. Mol Ecol.

[CR91] Sánchez-Montes S, Guzmán-Cornejo C, Martínez-Nájera Y, Becker I, Venzal JMJM, Labruna MB (2016). *Rickettsia lusitaniae* associated with *Ornithodoros yumatensis* (Acari: Argasidae) from two caves in Yucatan, Mexico. Ticks Tick Borne Dis.

[CR92] Sánchez RST, Santodomingo AMS, Muñoz-Leal S, Silva-de la Fuente MC, Llanos-Soto S, Salas LM, González-Acuña D (2020). Rodents as potential reservoirs for *Borrelia* spp. in northern Chile. Rev Bras Parasitol Veterinária.

[CR93] Sarwar M (2017). Status of argasid (soft) ticks (Acari: Parasitiformes: Argasidae) in relation to transmission of human pathogens. Int J Vaccines Vaccin.

[CR94] Scoles GA (2004). Phylogenetic analysis of the Francisella-like endosymbionts of dermacentor ticks. J Med Entomol.

[CR95] Shapiro ED, Gerber MA (2011) CHAPTER 17 - Borrelia infections: Lyme disease and relapsing fever. In: Remington JS, Klein JO, Wilson CB, Nizet V, Maldonado YABT-ID of the F. and N. (Seventh E. (eds), W.B. Saunders, Philadelphia, pp 564–576. 10.1016/B978-1-4160-6400-8.00017-1

[CR96] Shapiro MR, Fritz CL, Tait K, Paddock CD, Nicholson WL, Abramowicz KF, Karpathy SE, Dasch GA, Sumner JW, Adem PV, Scott JJ, Padgett KA, Zaki SR, Eremeeva ME (2010). Rickettsia 364D: a newly recognized cause of eschar-associated illness in California. Clin Infect Dis.

[CR97] Stich RW, Schaefer JJ, Bremer WG, Needham GR, Jittapalapong S (2008). Host surveys, ixodid tick biology and transmission scenarios as related to the tick-borne pathogen, *Ehrlichia canis*. Vet Parasitol.

[CR98] Subramanian G, Mediannikov O, Angelakis E, Socolovschi C, Kaplanski G, Martzolff L, Raoult D (2012). *Diplorickettsia massiliensis* as a human pathogen. Eur J Clin Microbiol Infect Dis.

[CR99] Sun LV, Scoles GA, Fish D, O’Neill SL (2000). Francisella-like endosymbionts of ticks. J Invertebr Pathol.

[CR100] Swei A, Kwan JY (2017). Tick microbiome and pathogen acquisition altered by host blood meal. ISME J.

[CR101] Tahir D, Socolovschi C, Marié J-L, Ganay G, Berenger J-M, Bompar J-M, Blanchet D, Cheuret M, Mediannikov O, Raoult D, Davoust B, Parola P (2016). New rickettsia species in soft ticks *Ornithodoros hasei* collected from bats in French Guiana. Ticks Tick Borne Dis.

[CR102] Tarquino-Carbonell A, del Gutiérrez-Díaz P, Galindo-Espinosa KA, Reinoso-Flórez EY, Solari G, Guerrero S (2015). Ectoparasites associated with bats in northeastern Tolima. Colombia Mastozoología Neotrop.

[CR103] Thoendel M (2020). Targeted metagenomics offers insights into potential tick-borne pathogens. J Clin Microbiol.

[CR104] Tijsse-Klasen E, Koopmans MPG, Sprong H (2014). Tick-borne pathogen–reversed and conventional discovery of disease. Front Public Health.

[CR105] Tokarz R, Lipkin WI (2021). Discovery and surveillance of tick-borne pathogens. J Med Entomol.

[CR106] Tufts DM, Sameroff S, Tagliafierro T, Jain K, Oleynik A, VanAcker MC, Diuk-Wasser MA, Lipkin WI, Tokarz R (2020). A metagenomic examination of the pathobiome of the invasive tick species, *Haemaphysalis longicornis*, collected from a New York City borough, USA. Ticks Tick Borne Dis.

[CR107] Vayssier-Taussat M, Kazimirova M, Hubalek Z, Hornok S, Farkas R, Cosson J-F, Bonnet S, Vourch G, Gasqui P, Mihalca AD, Plantard O, Silaghi C, Cutler S, Rizzoli A (2015). Emerging horizons for tick-borne pathogens: from the ``one pathogen-one disease’ vision to the pathobiome paradigm. Future Microbiol.

[CR108] Vilcins I-ME, Old JM, Deane E (2009). Molecular detection of *Rickettsia*, Coxiella and *Rickettsiella* DNA in three native Australian tick species. Exp Appl Acarol.

[CR109] Walker T, Johnson PH, Moreira LA, Iturbe-Ormaetxe I, Frentiu FD, McMeniman CJ, Leong YS, Dong Y, Axford J, Kriesner P (2011). The w Mel *Wolbachia* strain blocks dengue and invades caged *Aedes aegypti* populations. Nature.

[CR110] Wang G, Tang Y-W, Sussman M, Liu D, Poxton I, Schwartzman JB (2015). Chapter 104—*Borrelia burgdorferi* and other *Borrelia* species.

[CR111] Wechtaisong W, Bonnet SI, Lien Y-Y, Chuang S-T, Tsai Y-L (2020). Transmission of *Bartonella henselae* within *Rhipicephalus sanguineus*: data on the potential vector role of the tick. PLoS Negl Trop Dis.

[CR112] Wen TH, Chen Z (2016). The world list of ticks. 1. Argasidae and Nuttallielidae (Acari∶ Ixodida). J Parasitol Parasit Dis.

[CR113] Wu-Chuang A, Hodžić A, Mateos-Hernández L, Estrada-Peña A, Obregon D, Cabezas-Cruz A (2021). Current debates and advances in tick microbiome research. Curr Res Parasitol Vector-Borne Dis.

[CR114] Yan P, Qiu Z, Zhang T, Li Y, Wang W, Li M, Yu Z, Liu J (2019). Microbial diversity in the tick *Argas japonicus* (Acari: Argasidae) with a focus on *Rickettsia* pathogens. Med Vet Entomol.

[CR115] Zhang X-C, Yang Z-N, Lu B, Ma X-F, Zhang C-X, Xu H-J (2014). The composition and transmission of microbiome in hard tick, *Ixodes persulcatus*, during blood meal. Ticks Tick Borne Dis.

[CR116] Zhuang L, Du J, Cui X-M, Li H, Tang F, Zhang P-H, Hu J-G, Tong Y-G, Feng Z-C, Liu W (2018). Identification of tick-borne pathogen diversity by metagenomic analysis in *Haemaphysalis longicornis* from Xinyang, China. Infect Dis Poverty.

