# Supplementary material for: Bacteria related to tick-borne pathogen assemblages in Ornithodoros cf. hasei (Acari: Argasidae) and blood of the wild mammal hosts in the Orinoquia region, Colombia
Source: Exp Appl Acarol. 2022 Jul 13;87(2-3):253–71. doi: 10.1007/s10493-022-00724-9 (PMC9424158; doi:10.1007/s10493-022-00724-9)
Supplement: Supplementary file 1 — Supplementary material 1 (DOCX 18 kb) [file 10493_2022_724_MOESM1_ESM.docx]

**Table S1.** Collection information about wild mammals (Chiroptera) sampled and related to argasids of Arauca, Orinoquia region, Colombia.

| **Museum Code** | **Bat species** | **Number of ticks** | **Municipality** | **Locality** | **Elevation (m)** | **Coordinates** |
| --- | --- | --- | --- | --- | --- | --- |
| MHN-UCa-M 2317 | *Cynomops planirostrisΦ* | 1 Larva | Arauca | Vereda Las Plumas, Sitio Los Cunaguaros | 112 | 06°36'15" N 70°29'52" W |
| MHN-UCa-M 2265 | *Noctilio albiventris* | 5 Larvae | Cravo Norte | Vereda El Deleite | 111 | 06°32'25.2" N 70°31'23.6" W |
| MHN-UCa-M 2828 | *Cynomops planirostrisΦ* | 8 Larvae | Arauca | Vereda El Socorro, Finca Los Trompillos | 134 | 06°47'3.23"N 70°42'8.2"W |
| MHN-UCa-M 2814 | *Molossus pretiosusΦ* | 2 Larvae | Arauca | Vereda El Socorro, Finca Los Trompillos | 134 | 06°47'3.23"N 70°42'8.2"W |
| MHN-UCa-M 2262 | *Noctilio albiventris* | 2 Larvae | Cravo Norte | Vereda El Deleite | 111 | 06°32'25.2" N 70°31'23.6" W |
| MHN-UCa-M 2253 | *Noctilio albiventris* | 8 Larvae | Arauca | Vereda Las Plumas, Sector Guayabital | 123 | 06°37'29.4" N 70°35'9.9" W |
| MHN-UCa-M 2323 | *Cynomops planirostrisΦ* | 1 Larva | Arauca | Vereda Las Plumas, Sitio Los Cunaguaros | 112 | 06°36'15" N 70°29'52" W |
| MHN-UCa-M 2327 | *Cynomops planirostrisΦ* | 1 Larva | Cravo Norte | Vereda El Deleite | 111 | 06°32'25.2" N 70°31'23.6" W |
| MHN-UCa-M 2328 | *Cynomops planirostrisΦ* | 1 Larva | Cravo Norte | Vereda El Deleite | 111 | 06°32'25.2" N 70°31'23.6" W |
| MHN-UCa-M 2928 | *Myotis handleyiΦ* | 7 Larvae | Arauca | Vereda Las Plumas, Finca Las Piñas | 110 | 06°35'16.7" N 70°30'9.8" W |
| MHN-UCa-M 2869 | *Molossus pretiosusΦ* | 2 Larvae | Arauca | Vereda El Socorro, Finca Los Trompillos | 134 | 06°47'3.23"N 70°42'8.2"W |
| MHN-UCa-M 2806 | *Noctilio albiventris* | (5 Nymphs, 104 Larvae) | Arauca | Vereda El Socorro, Finca Los Trompillos | 134 | 06°47'3.23"N 70°42'8.2"W |
| MHN-UCa-M 2822 | *Molossus pretiosusΦ* | 14 Larvae | Arauca | Vereda El Socorro, Finca Los Trompillos | 134 | 06°47'3.23"N 70°42'8.2"W |
| MHN-UCa-M 2808 | *Molossus pretiosusΦ* | 1 Larva | Arauca | Vereda El Socorro, Finca Los Trompillos | 134 | 06°47'3.23"N 70°42'8.2"W |
| MHN-UCa-M 2934 | *Molossus pretiosusΦ* | (1 Larva, 1 Nymph) | Arauca | Vereda El Socorro, Finca Los Trompillos | 134 | 06°47'3.23"N 70°42'8.2"W |
| MHN-UCa-M 2863 | *Molossus pretiosusΦ* | 2 Larvae | Arauca | Vereda El Socorro, Finca Los Trompillos | 134 | 06°47'3.23"N 70°42'8.2"W |
| MHN-UCa-M 2864 | *Molossus pretiosusΦ* | 1 Larva | Arauca | Vereda El Socorro, Finca Los Trompillos | 134 | 06°47'3.23"N 70°42'8.2"W |
| MHN-UCa-M 2809 | *Molossus pretiosusΦ* | 1 Larva | Arauca | Vereda El Socorro, Finca Los Trompillos | 134 | 06°47'3.23"N 70°42'8.2"W |
| MHN-UCa-M 2856 | *Molossus pretiosusΦ* | 1 Larva | Arauca | Vereda El Socorro, Finca Los Trompillos | 134 | 06°47'3.23"N 70°42'8.2"W |

*Φ* Associations of *O.* cf. *hasei* with mammal species are described for the first time in this study.
